# Uncertainty estimation for reliable neural network-based radiotherapy dose modelling using Monte Carlo dropout, mean variance estimation and deep ensemble^☆^

**DOI:** 10.1016/j.phro.2026.101072

**Published:** 2026-08-31

**Authors:** Moritz Schneider, Tabea Eberhardt, Cihan Gani, Paul Fischer, Christian F. Baumgartner, Daniela Thorwarth

**Affiliations:** a Section for Biomedical Physics, Department of Radiation Oncology, University Hospital Tübingen, Tübingen, Germany; b Department of Radiation Oncology, University Hospital Tübingen, Tübingen, Germany; c Cluster of Excellence “Machine Learning: New Perspectives for Science”, University of Tübingen, Germany; d Department of Biomedical Engineering, University of Basel, Switzerland; e Faculty of Health Sciences and Medicine, University of Lucerne, Switzerland; f German Cancer Consortium (DKTK), partner site Tübingen, a partnership between DKFZ and University Hospital Tübingen, Germany

**Keywords:** Dose modelling, Uncertainty estimation, Deep learning, Artificial intelligence, Dose calculation, Radiotherapy, MRgRT

## Abstract

**Background and Purpose:**

Neural networks promise fast dose modelling with high accuracy for challenging situations like magnetic resonance imaging (MRI)-guided radiotherapy. As they are data-driven, failure can occur and early identification of erroneous dose calculations is required.

In this study, we implemented and evaluated three uncertainty estimation techniques to assess whether they can indicate increased prediction error.

**Materials and methods:**

Using a dataset of 6713 radiotherapy segments from 130 1.5 T MRI linear accelerator plans and a 3D UNet for dose modelling, three techniques were implemented to assess uncertainty: Monte Carlo dropout (MCD), mean variance estimation (MVE) and a deep ensemble (DE).

All methods were evaluated regarding calibration using the expected normalized calibration error (ENCE) and correlation to mean absolute error (MAE).

Cumulative failure rates were calculated across uncertainty thresholds, with a 3 mm/3% gamma passing rate < 95% defining failure.

**Results:**

After calibration, all three methods showed ENCE values of 0.14/0.05/0.10 for MCD/MVE/DE. A strong relationship between mean uncertainty and MAE was observed, reflected by high Spearman correlation coefficients (MCD: ρ = 0.64, MVE: ρ = 0.76, DE: ρ = 0.67).

Failure rates increased with mean uncertainty across all methods, enabling thresholds targeting a 10% failure rate. On independent evaluation, 44%–77% of segments were accepted, with observed failure rates among accepted segments of 7.2%–9.5%.

**Conclusions:**

All methods provided meaningful uncertainty estimates clearly associated with prediction error. MVE showed the lowest ENCE and strongest correlation with MAE while requiring the least computation. DE most closely matched the target failure rate while flagging the fewest segments.

## Highlights

• All methods yielded informative uncertainty with low calibration error (0.05–0.14).
• Uncertainty strongly correlated with mean absolute error per segment (ρ = 0.64–0.76).
• Risk of failing clinical criterion steadily increased with higher uncertainty.
• Thresholding accepted 44%–77% of segments with gamma failure below the 10% target.
• Mean variance estimation provided uncertainty information with single forward pass.

## Graphical abstract

**Figure:**
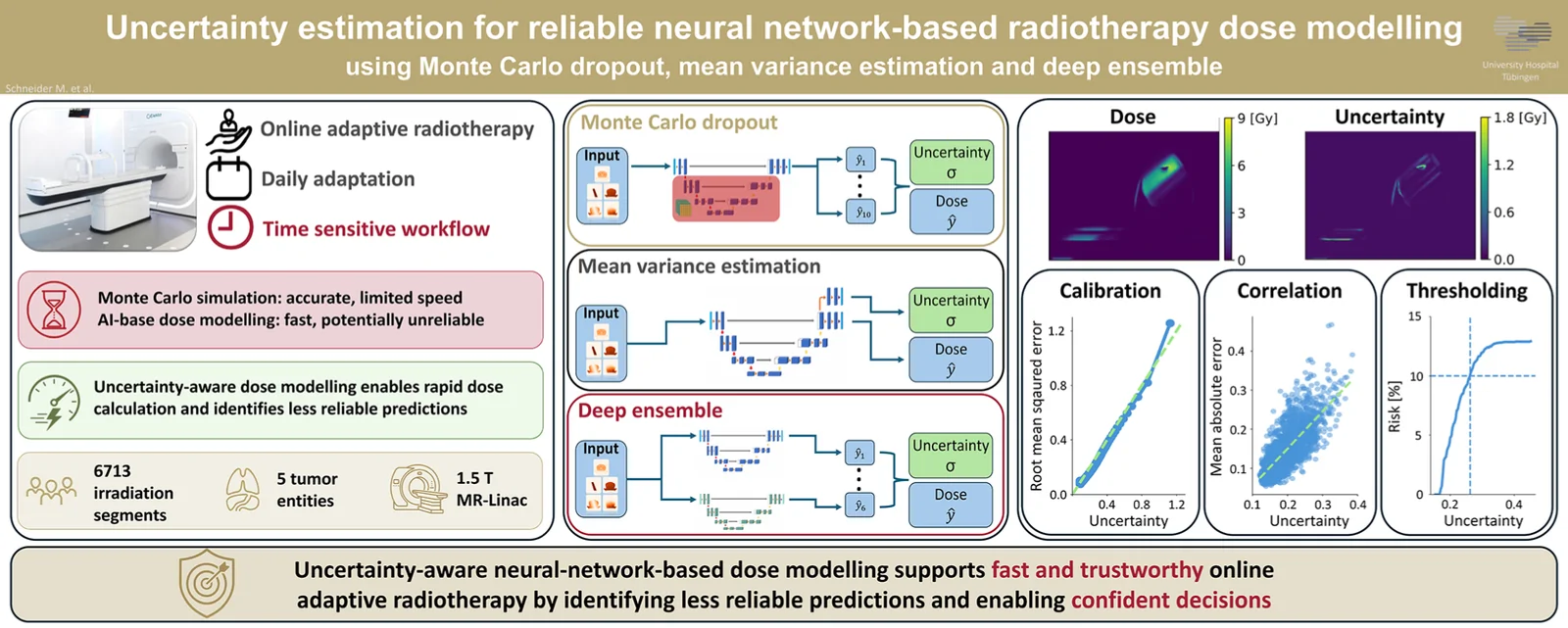

## 1 Introduction

In recent years, on-table imaging modalities showed increasing impact on radiotherapy [1], [2], [3]. With improved image quality, strategies to assign electron densities and fast structure propagation techniques, adaption of the treatment plan to the current anatomical situation got feasible [4], [5], [6]. Online plan adaptation aims to exploit anatomical information acquired immediately prior or during treatment delivery to reduce the risks associated with inter- and intrafraction motion and ensure the intended target coverage and organ at risk sparing [7], [8]. To fully leverage its potential, time associated risks should be minimized by a fast workflow [9]. Therefore, emerging techniques like neural networks are used to streamline and automate time critical workflow steps like plan adaptation [10], [11], [12] or organ segmentation [13], [14].

Iterative calculation of deposited dose during the optimization step of plan adaptation is a further computational bottleneck [15]. Monte Carlo (MC) simulated dose distributions are the current gold standard in dose calculation, offering precise physics-based simulation, even for challenging situations like magnetic resonance guided radiotherapy (MRgRT) [16], [17]. But as high accuracy in MC simulations requires high numbers of incident particles, it comes at the cost of computational resources and time [15], [18]. While present implementations of MC dose engines using graphics processing units (GPU) require computation times in order of minutes [17], [19], [20], neural networks only require seconds to sub-seconds of reported inference time for comparable dose calculation tasks [21], [22], [23], [24].

Nevertheless, these approaches are data-driven and do not explicitly encode physical laws, leaving the risk of failure due to a distribution shift, residual errors or out of distribution samples [25], [26]. Therefore, especially in high-risk applications like radiotherapy, quantification of the prediction uncertainty is crucial for a safe integration of these methods into clinical workflows. Since auto-segmentation is the most common neural network application in radiatiotherapy, several studies have investigated uncertainty estimation in this field [27], [28], [29]. Uncertainty estimations could identify structures and regions with low confidence and guide reviewer attention [30]. Rabe et al. [31] calibrated uncertainty at the patient level to provide quantitative estimates rather than qualitative maps.

In the area of dose modelling only very few approaches for uncertainty estimation are present, not covering applicability of the created uncertainty measures. Fischer et al. [32] demonstrated the use of conformal prediction for uncertainty estimation in dose modelling, but only evaluated the validity of the calibration.

The aim of this work was to close this gap by evaluating three approaches for uncertainty quantification, deep ensembles, Monte Carlo dropout and mean variance estimation to enable meaningful and well calibrated uncertainty information together with their potential use in MRgRT dose modelling.

## 2 Materials and methods

### 2.1 Patient data

For this work, the same dataset was used as in the training of the previously published base model [23]. It consists of intensity modulated radiotherapy (IMRT) plan data and computed tomography images of 130 patients treated at a 1.5 T MRI linear accelerator (Unity, Elekta AB, Sweden). The 6713 irradiation segments were split into training and test dataset, consisting of 3956 segments from 75 patients and 2374 irradiation segments from 45 patients, respectively. To enable a valid calibration of the estimated uncertainty, 383 irradiation segments from 10 patients were held out for subsequent calibration. The dataset consisted of IMRT plans from prostate, liver and head and neck cancer (HNC) patients, as well as partial breast irradiation and tumorous lymph nodes.

Full MC-simulations using EGSnrc [33] served as ground-truth, using *n* = 5E07 primary particles per segment and a dose grid voxel size of 3x3x3 mm^3^.

### 2.2 Implementation of uncertainty estimation

**Fig. 1:**
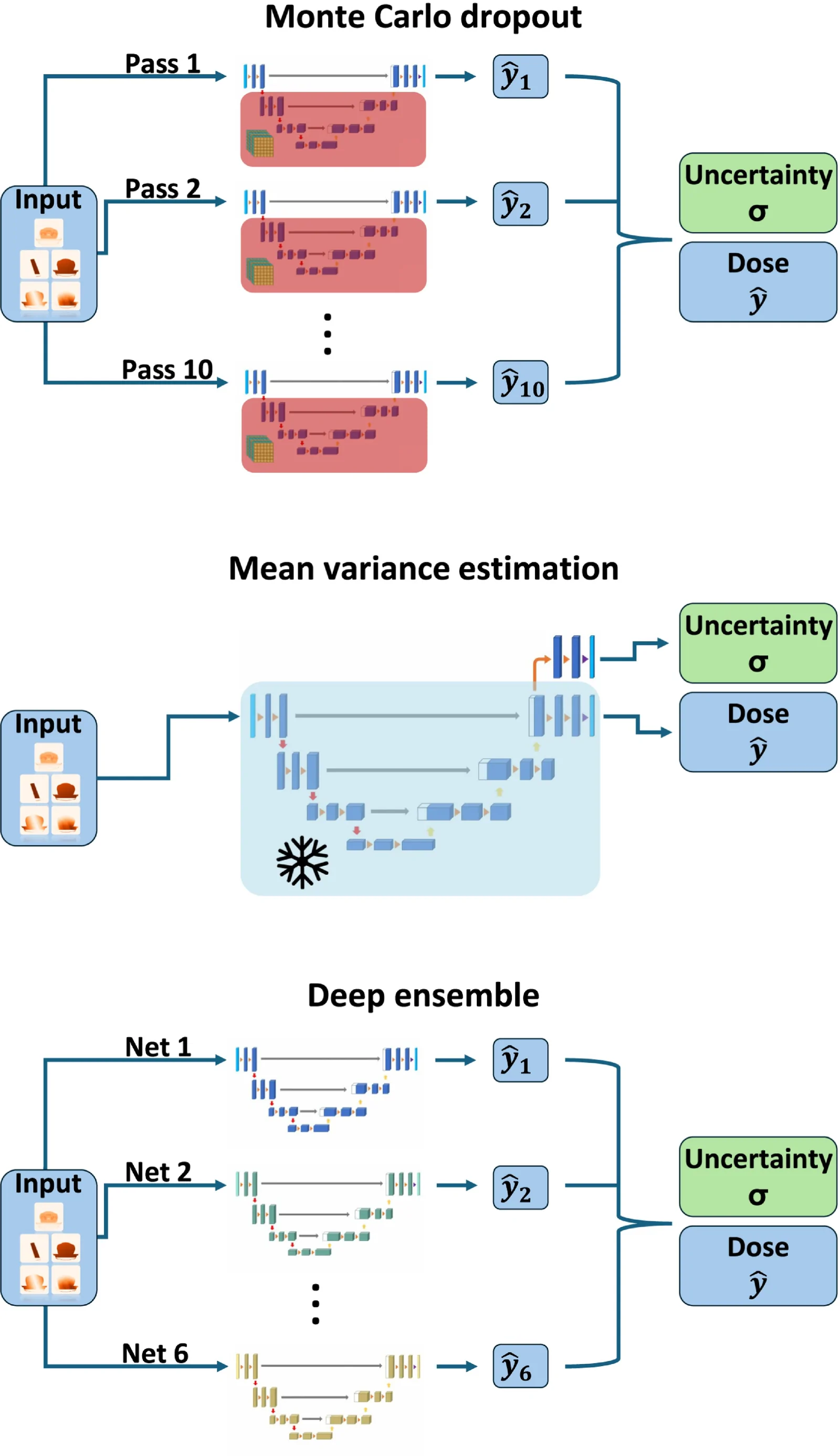
**Overview on network architectures used in this work**. Monte Carlo dropout (top) with ten forward passes and feature wise dropout in all but the first layer, mean variance estimation (middle) with fixed base network and separately trained uncertainty output and deep ensemble (bottom) with six individually trained base networks.

The architecture described in [23], which is based on a 3D UNet with five physically meaningful input masks, was used as starting point. Three different uncertainty estimation methods were implemented, as outlined in the following (Fig. 1).

**Monte Carlo dropout** (MCD) was implemented as spatial dropout disabling entire feature maps, as described in [34], [35]. No dropout was applied to the highest level of the 3D UNet, as otherwise the uncertainty estimation poorly reflected the prediction error. With a dropout rate of 0.1, the model was inferred ten times, delivering the MCD prediction as the arithmetic mean and the uncertainty as the standard deviation of the individual outputs.

In case of the **deep ensemble** (DE), the ensemble members shared the same network architecture and training data but differed in their random weight initialization. Three models were trained using the root mean squared error (RMSE) loss and three models were trained using the customized loss function described in [23] to promote model diversity. The ensemble prediction is obtained by taking the arithmetic mean of the individual model outputs. The uncertainty is then calculated as its standard deviation.

To implement the **mean variance estimation** (MVE) method, a second model output was added to the base model to estimate the logarithm of the variance, as suggested by Kendall et al. [36]. The model from the DE with the lowest loss in the validation set was used as a pretraining, optimized for the dose output. The weights of this model were fixed and in a second training step only the uncertainty output was optimized.

### 2.3 Calibration

The predicted uncertainty was calibrated separately for each uncertainty estimation method using the dedicated calibration subset. Voxel-wise predictions with a target dose exceeding 1% of the segment-specific maximum dose were retained and eligible voxels were pooled. The uncertainty values were sorted into 100 quantile bins. For each bin, the mean predicted uncertainty and the RMSE between the predicted and reference dose were calculated. A monotonic isotonic regression was then fitted and applied to the uncertainty predictions of the independent test subset without adjustment.

Confidence intervals were estimated using patient-level bootstrap resampling. For each uncertainty estimation method, 1000 bootstrap samples were generated by sampling patients from the test dataset with replacement. The calibration function fitted on the calibration subset were kept fixed throughout bootstrapping.

### 2.4 Evaluation

The consistency of the modelled dose with the ground truth was evaluated for all three approaches using a 3 mm/3% gamma criterion with a 10% dose cutoff.

The connection between estimated uncertainty and observed error was evaluated on the test dataset using reliability diagrams and the expected normalized calibration error (ENCE) before and after calibration. In order to assess the uncertainty of the calibration error, 95% confidence intervals for the reliability curves and 95% confidence intervals for the ENCE were determined from the bootstrap distributions.

Correlation of uncertainty histogram properties with mean absolute error (MAE) and gamma passing rate were examined using Spearman correlation coefficient (ρ). Properties tested included mean uncertainty, median uncertainty, the 90th percentile of the uncertainty distribution and the interquartile range of the uncertainty distribution. They were correlated with the mean absolute error in areas with the dose being higher than 1% of the maximum dose, the 3 mm/3% gamma criterion and the 1 mm/1% gamma criterion, both with dose cutoff of 10%. The latter two were used to connect estimated model uncertainty to metrics used in clinical practice.

To investigate whether uncertainty estimates differed systematically between tumor sites, an entity-wise subgroup analysis was performed. Segment-level mean uncertainty values were grouped according to the tumor site. All segments originating from the same patient were aggregated using the mean of the segment-level uncertainty. This patient-level data was used for a Kruskal-Wallis-based permutation test with Bonferroni-Holm *p*-value correction.

To define and independently evaluate an uncertainty threshold for flagging segments at increased risk of dose-modelling error, the test dataset was split at the patient level while maintaining comparable entity distributions in both subsets. All patients from the calibration dataset were then added to one half of the test dataset to form the threshold-definition set, while the remaining test patients constituted the independent threshold-evaluation set. The resulting datasets contained 1510 and 1247 segments, respectively. In the threshold-definition set, segments were ordered by their mean uncertainty and the observed proportion of segments with a gamma passing rate below 95% at 3 mm/3% was determined as a function of the uncertainty threshold. The uncertainty value corresponding to a 10% failure rate among accepted segments was selected and applied unchanged to the independent threshold-evaluation set. The proportions accepted segments, the failure rate among accepted segments, sensitivity, specificity, positive predictive value and negative predictive value were compared across the three uncertainty estimation methods.

## 3 Results

All three models achieved comparable agreement with the ground truth full MC simulation. With a 3 mm/3% gamma criterion, the network with MCD exhibited a mean passing rate of 97.4% with standard deviation of 2.8% over all segments in the test dataset. MVE and DE showed mean (standard deviation) on segment level of 97.8% (2.8%) and 97.6% (2.7%). With that, implementation of the uncertainty estimation did not introduce any significant changes compared to the base network.

**Fig. 2:**
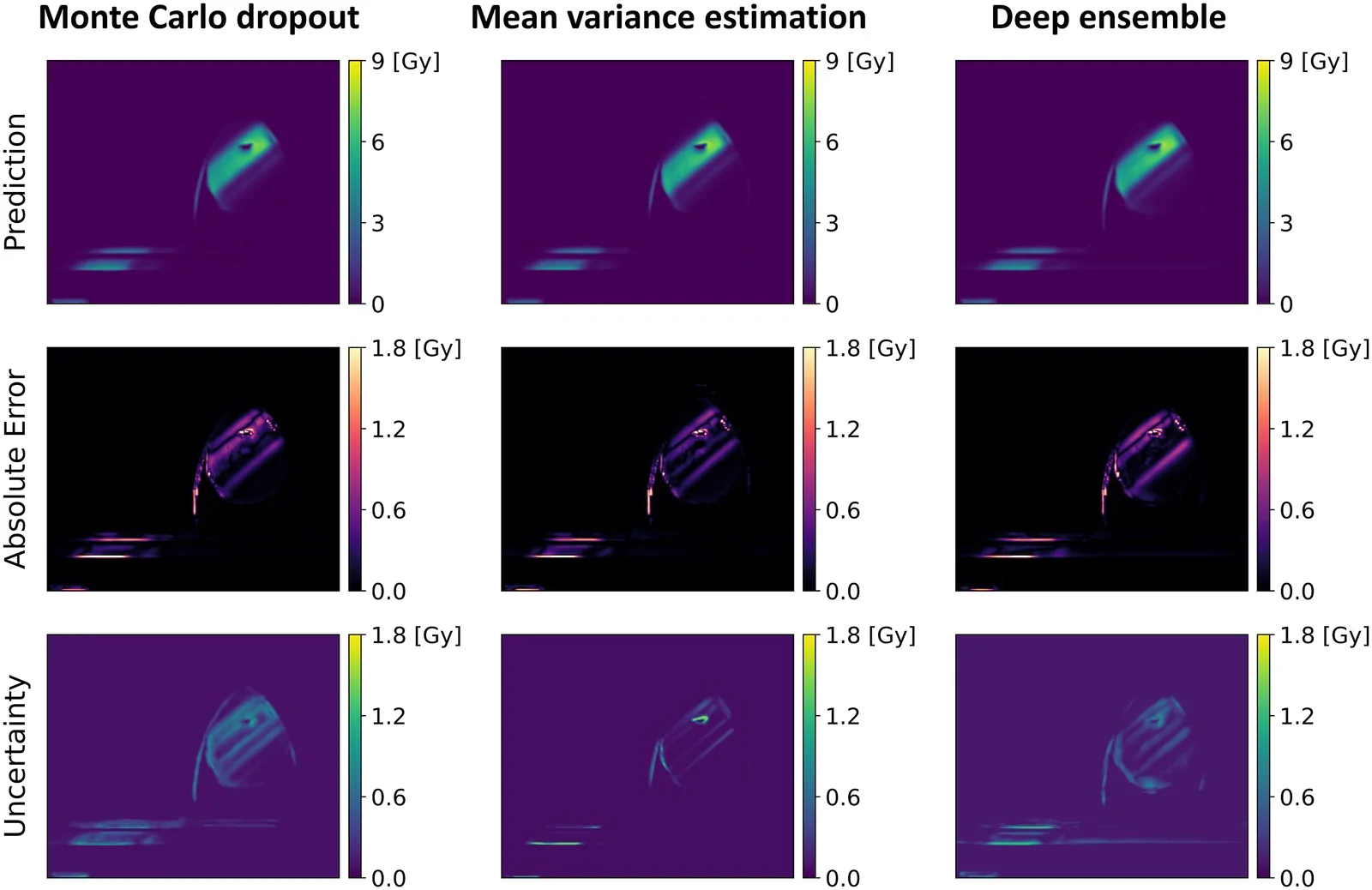
**Qualitative example** of modelled dose, calibrated uncertainty and absolute error for all three methods used in this work.

All three approaches produced meaningful spatial uncertainty estimates on the test set. Elevated uncertainty was primarily observed in regions with steep dose gradients, such as field edges or air cavities and visually corresponded to areas of increased prediction error (Fig. 2). Artifacts outside the beam could not be observed.

**Fig. 3:**
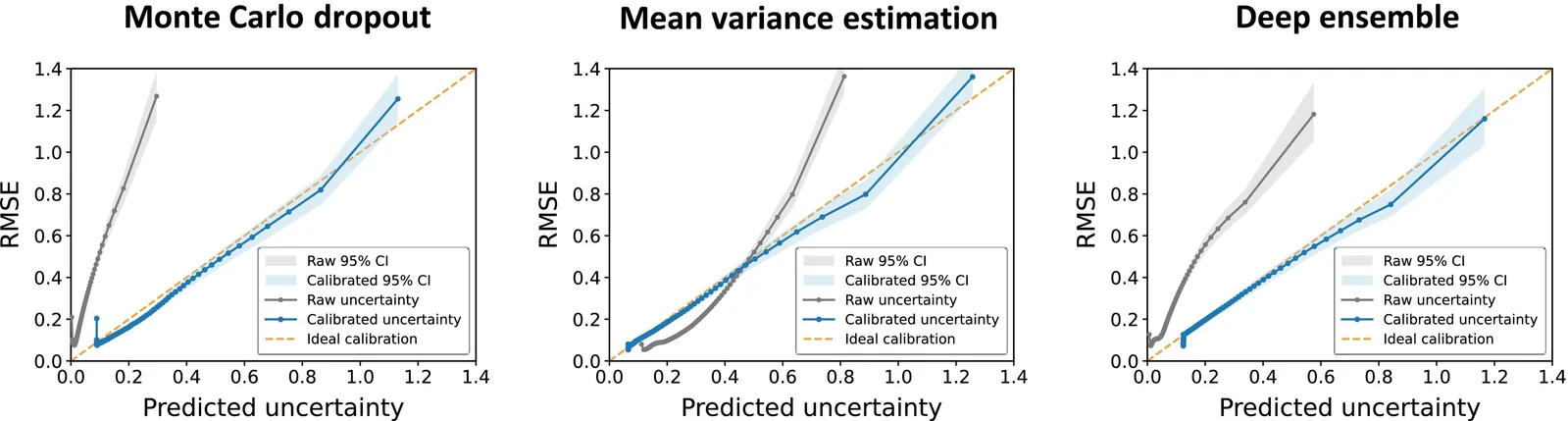
**Reliability of uncertainty estimates before (grey) and after (blue) calibration.** Reliability plots compare binned predicted uncertainty with the corresponding root mean squared error (RMSE) for MCD, MVE, and DE on the test set. Raw and calibrated uncertainty estimates are shown with patient-level bootstrap 95% confidence intervals (CI). Ideal calibration is presented in orange. (For interpretation of the references to colour in this figure legend, the reader is referred to the web version of this article.)

Before calibration, MCD and DE exhibited a high ENCE of 6.57 (95% bootstrap confidence interval, 5.98–7.12) and 2.37 (2.04–2.70), whereas for MVE the ENCE was 0.42 (0.39–0.45) on the test dataset. After calibration, predicted uncertainty exhibited a strong linear relationship with the observed prediction error (Fig. 3). The ENCE (95% confidence interval) after calibration was 0.14 (0.09–0.20) for MCD, 0.05 (0.03–0.09) for MVE and 0.10 (0.08–0.18) for the DE. At low predicted uncertainty levels, DE tended to produce conservative uncertainty estimates, whereas MCD exhibited overconfident behavior. MVE showed the best correspondence between uncertainty and root mean squared error and therefore the lowest calibration error.

For all three methods, the mean uncertainty per segment showed the strongest correlation with the mean absolute error (Table 1). The Spearman correlation coefficient between mean uncertainty and mean absolute error was 0.64 for MCD, 0.76 for MVE and 0.67 for DE.

**Table 1:** Spearman correlation coefficient (ρ) for uncertainty measures and estimation methods.

	Monte Carlo dropout			Mean variance estimation			Deep ensemble		
	MAE	3%/3 mm	1%/ 1 mm	MAE	3%/3 mm	1%/ 1 mm	MAE	3%/3 mm	1%/ 1 mm
Mean uncertainty	0.64	−0.38	−0.38	0.76	−0.44	−0.35	0.67	−0.41	−0.52
Median uncertainty	0.60	−0.31	−0.30	0.65	−0.37	−0.27	0.58	−0.34	−0.46
P90	0.62	−0.40	−0.41	0.74	−0.45	−0.36	0.65	−0.41	−0.53
Inter-quartile-range	0.61	−0.41	−0.41	0.71	−0.48	−0.39	0.67	−0.41	−0.54

Correlations with gamma-based metrics were generally weaker, which is consistent with the bounded range of gamma passing rates and the spatial tolerance incorporated in the gamma evaluation (Table 1). For the 3 mm/3% and 1 mm/1% gamma criteria, the Spearman correlation coefficients between mean uncertainty and the corresponding gamma passing rates were − 0.38 and − 0.38, −0.44 and − 0.35, −0.41 and − 0.52 for MCD, MVE and DE, respectively. The distribution of uncertainty and agreement metrics further illustrates this relationship. Most segments exhibited low mean uncertainty and high agreement with the ground truth, whereas higher uncertainty levels were increasingly associated with reduced agreement (Supplementary fig. S1).

**Fig. 4:**
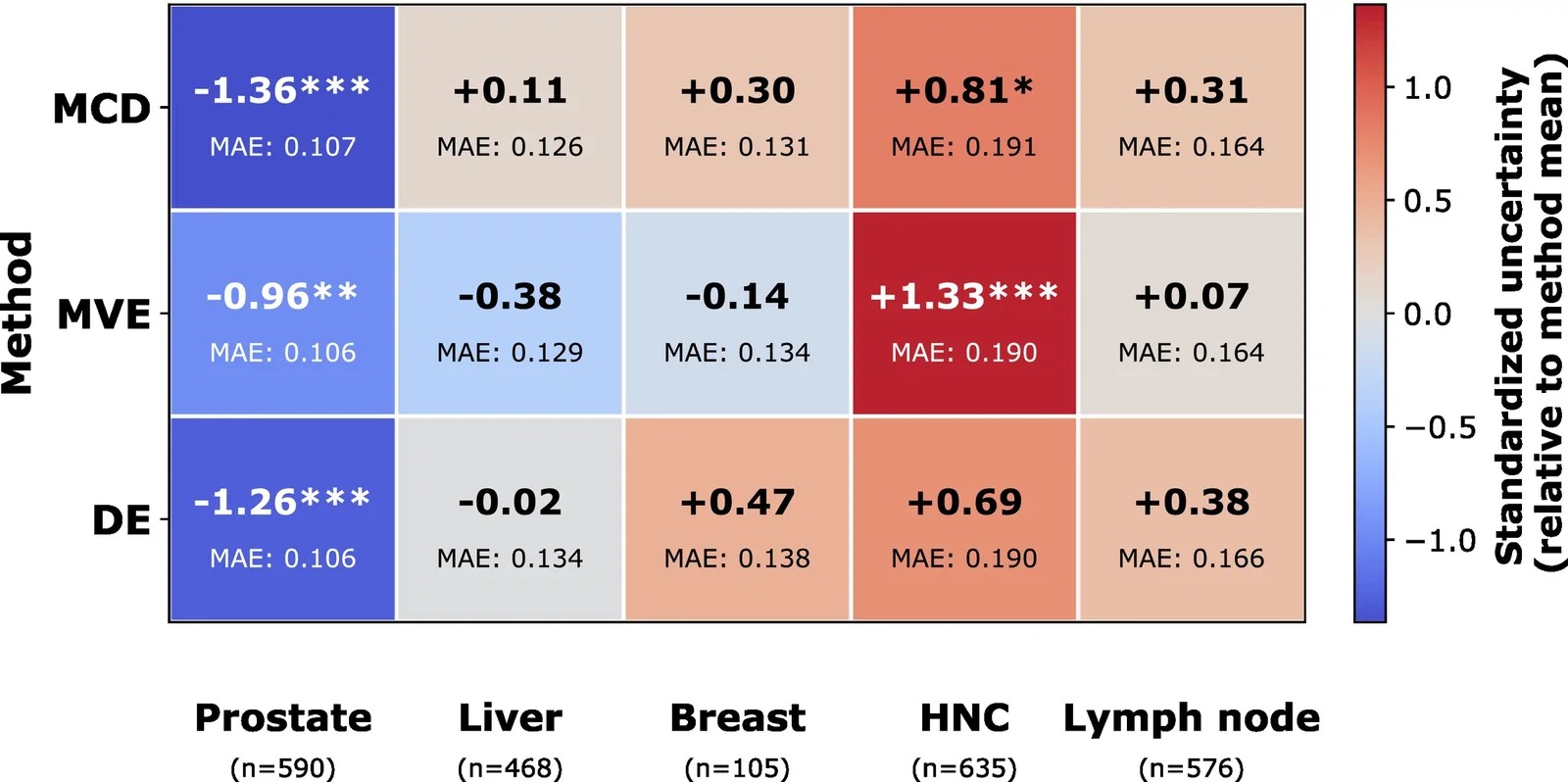
**Tumor entity–specific uncertainty and prediction error at the patient level.** The heatmap shows standardized mean patient-level uncertainty together with mean absolute error (MAE), for each tumor entity and method. Segment-level values were averaged per patient, and uncertainty was z-standardized within each method across entities. Significance markers indicate patient-level permutation tests based on Bonferroni-Holm corrected Kruskal–Wallis H statistic, comparing each entity with the remaining entities (*p < 0.05, **p < 0.01, ***p < 0.001).

Analyzing the different tumor entities separately, patient-level uncertainty was significantly lower for prostate than for the remaining tumor sites across all three methods (*p* < 0.001, *p* = 0.003, *p* < 0.001 for MCD, MVE and DE respectively). In contrast, head-and-neck cancer dose distributions showed significantly higher uncertainty for MCD and MVE (*p* = 0.015, *p* < 0.001). This pattern was mirrored by the MAE, which was lowest for prostate (0.106–0.107) and highest for HNC cancer (0.190–0.191) across the three methods (Fig. 4).

**Fig. 5:**
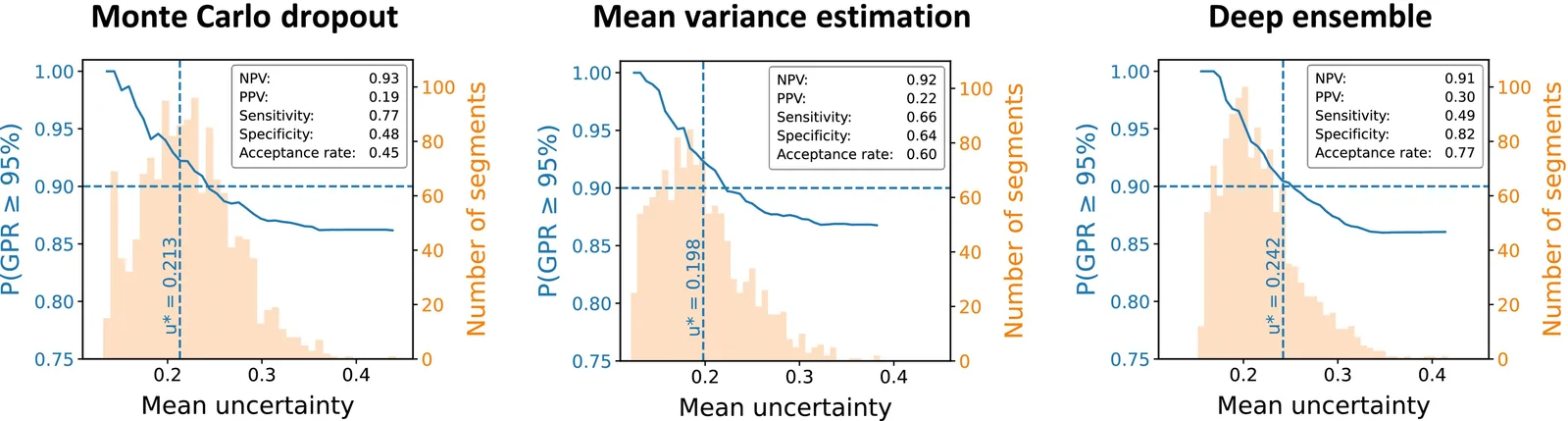
**Independent evaluation of uncertainty-based thresholds.** The predefined threshold for each method was applied to the independent evaluation set. The curve shows the proportion of segments below a given mean-uncertainty value u that met the 3 mm/3% gamma criterion (GPR ≥95%). The vertical dashed line marks the predefined uncertainty threshold, while the horizontal dashed line marks the 90% target, equivalent to a 10% failure rate among accepted segments. Histograms show the distribution of mean uncertainty, and the inset summarizes sensitivity, specificity, positive and negative predictive values, and acceptance rate at the selected threshold.

In the threshold-definition set, the cumulative proportion of segments with a 3 mm/3% gamma passing rate ≥ 95% decreased with increasing mean uncertainty for all methods. Thresholds corresponding to a 10% failure rate among accepted segments, equivalent to a negative predictive value (NPV) of 90%, were 0.213 for MCD, 0.198 for MVE and 0.242 for DE. In the independent evaluation subset, the relationship between segment-level uncertainty and failure rate (Fig. 5) was similar to that observed in the threshold-definition subset. Application of the previously defined thresholds on the threshold-evaluation subset resulted in failure rates among accepted segments of 7.2%, 7.5% and 9.1% for MCD, MVE and DE. Thus, the failure rate was below the targeted 10% for all methods. The MCD threshold achieved the highest sensitivity for detecting failing segments (0.77), but accepted only 45% of all segments, resulting in more than half of the segments being flagged and the lowest specificity of 0.48. MVE achieved a higher acceptance rate (0.60) and specificity (0.64), but a lower sensitivity (0.66). DE most closely matched the intended operating point, achieved the highest acceptance rate (0.77) and specificity (0.82) and the lowest sensitivity of 0.49. The positive predictive values were 0.19, 0.22 and 0.30 for MCD, MVE and DE, respectively (Fig. 5). Accordingly, the observed failure rate among flagged segments was 2.6-, 2.9- and 3.2-fold higher than that among accepted segments.

## 4 Discussion

In this work, a previously presented dose modelling network for MRgRT [23] was extended with three uncertainty estimation methods, yielding low post-calibration ENCE values of 0.05–0.14 and a strong segment-level correlation between mean uncertainty and prediction error. Based on this association, a threshold on segment- level mean uncertainty was determined to flag irradiation segments at elevated risk of insufficient agreement and was subsequently evaluated on an independent dataset. With an acceptance rate of up to 77%, this approach may improve efficiency in time sensitive application scenarios.

Differences between the three methods inherently arose from their implementations. In case of DE, several models had to be trained and evaluated during inference. Using MCD, only one model was required to be trained, but this model needed to be evaluated several times with random dropout. In contrast, MVE estimated the uncertainty using a dedicated output head. Consequently, the estimated uncertainty reflected a learned heteroscedastic error model rather than stochastic variation or disagreement between independently trained models. The main practical advantage of MVE was that only a single network needed to be trained and a single forward pass was required at inference time. MVE not only reduced the computational costs during training, but also in the time sensitive regime of inference. It resulted in a negligible increase in inference time compared with the base model, whereas DE and MCD required approximately sixfold and tenfold higher computational costs, respectively. As one of the main reasons for deep learning-based dose modelling is the reduction of calculation time e.g. in an online adaptive setting, this aspect is highly important [17], [37]. Despite its substantially lower computational requirements, MVE showed advantages in calibration and correlation between uncertainty and prediction error.

Entity specific agreement between uncertainty and MAE, especially in prostate and HNC patient cases, suggested that the estimates reflected differences in prediction difficulty. This also applied to the unseen lymph-node entity. This agreement is encouraging, as the uncertainty estimates appeared to reflect the increased prediction error for an unseen entity rather than remaining inappropriately low. Further out-of-distribution validation is required to determine whether this behavior generalizes.

For all three methods, the cumulative proportion of segments with a 3 mm/3% gamma passing rate below 95% increased with segment-level mean uncertainty. This supports the use of an uncertainty threshold to maintain the failure rate below 10%. When applied to the independent test set, the predefined thresholds were more conservative than intended for all three methods but remained close to the target operating point, indicating reasonable transferability to unseen data. The observed deviation may partly have reflected sampling variability due to the limited dataset size. DE showed the closest agreement with the intended operating point and the most selective enrichment of high-risk cases. However, prospective validation using a cohort reflecting the intended clinical population and the specific application scenario is required before clinical implementation.

The low ENCE, the strong correlation between uncertainty and error metrics and the increasing failure rate with uncertainty suggested that the estimated uncertainty provided relevant information about prediction reliability. Accordingly, uncertainty estimates may help identify cases at increased risk of large prediction errors or failure of a predefined quality criterion and could therefore support targeted review workflows.

Flagged segments could be redirected towards a MC simulation in order to maintain the given risk criteria, while leveraging the high-speed potential of neural network-based dose modelling in many cases. Alternatively, a plan-level global uncertainty measure or the proportion of flagged segments could trigger MC recalculation of all segments originating from respective treatment plans.

The recent review of Duenger et al. [26] emphasized that uncertainties are inherent to radiotherapy models, that their quantification is essential for transparent and reliable predictions and that uncertainty estimates may support clinical translation and quality assurance workflows. To our knowledge, there is still very limited literature on the use of uncertainty estimation for neural network-based dose modelling and how uncertainty propagates when aggregated into a single segment-level quantity. In their review, Wahid et al. [29] described that most uncertainty estimates are targeted on segmentation and noted that problems represented only at the patient level are intrinsically harder than tasks with more granular image-level information. Existing dose-prediction studies, which is the area with closest relation to dose modelling, tend to quantify uncertainty on the predicted dose level rather than on aggregated components. Nguyen et al. defined uncertainty as the voxel-wise standard deviation across stochastic predictions and reported Pearson correlations between uncertainty and absolute error of 0.55 in the body–PTV region and 0.27 in the PTV for bagging, while Tan et al. found that uncertainty was highly correlated with prediction errors, exhibiting Spearman correlation coefficients between 0.62 and 0.69 with DE performing better than MCD. For presenting uncertainty to the user, they both focused on uncertainty heatmaps and DVH confidence intervals not condensing the uncertainty in a single measure [38], [39]. These studies support the interpretation that uncertainty is most naturally preserved and validated at the local level, whereas robust aggregation into a single global uncertainty measure remains underdeveloped.

In conclusion we successfully added meaningful uncertainty estimation to our dose modelling framework and enabled clinical decision making based on three different approaches. While MVE comes with the lowest computational burden, exhibited the best calibration result and showed the strongest correlation with the mean absolute error, DE ensures the highest acceptance rate when using an uncertainty threshold for flagging segments with an elevated risk for low agreement with the ground truth. The use of uncertainty estimation in high-risk radiotherapy applications may improve confidence in model predictions, facilitate clinical decision making and support the safe clinical integration of neural networks.

## Declaration of generative AI and AI-assisted technologies in the manuscript preparation process

During the preparation of this work the author(s) used ChatGPT in order to refine phrasing. After using this tool/service, the author(s) reviewed and edited the content as needed and take(s) full responsibility for the content of the published article.

## CRediT authorship contribution statement

**Moritz Schneider:** Writing – original draft, Visualization, Software, Methodology, Investigation, Formal analysis. **Tabea Eberhardt:** Writing – review & editing, Software, Methodology, Investigation. **Cihan Gani:** Writing – review & editing, Resources. **Paul Fischer:** Writing – review & editing, Methodology. **Christian F. Baumgartner:** Writing – review & editing, Supervision, Resources, Methodology. **Daniela Thorwarth:** Writing – review & editing, Supervision, Resources, Conceptualization.

## Declaration of competing interest

The authorsdeclare the following financial interests/personal relationships which may be considered as potential competing interests: Funding: DFG (ZI 736/2-1). COI: research collaborations Elekta AB, Philips, PTW Freiburg, Dr. Sennewald Medizintechnik, Kaiku Health, TheraPanacea, ITV, Brainlab. Cihan Gani received travel and speaker honoraria from Elekta.
