# Supplementary material for: Uncertainty estimation for reliable neural network-based radiotherapy dose modelling using Monte Carlo dropout, mean variance estimation and deep ensemble
Source: Phys Imaging Radiat Oncol. 2026 Aug 31;41:101072. doi: 10.1016/j.phro.2026.101072 (PMC13572023; doi:10.1016/j.phro.2026.101072)
Supplement: Supplementary file 1 [file mmc1.pdf]

Supplementary material

A Correlation of mean uncertainty and error metrices

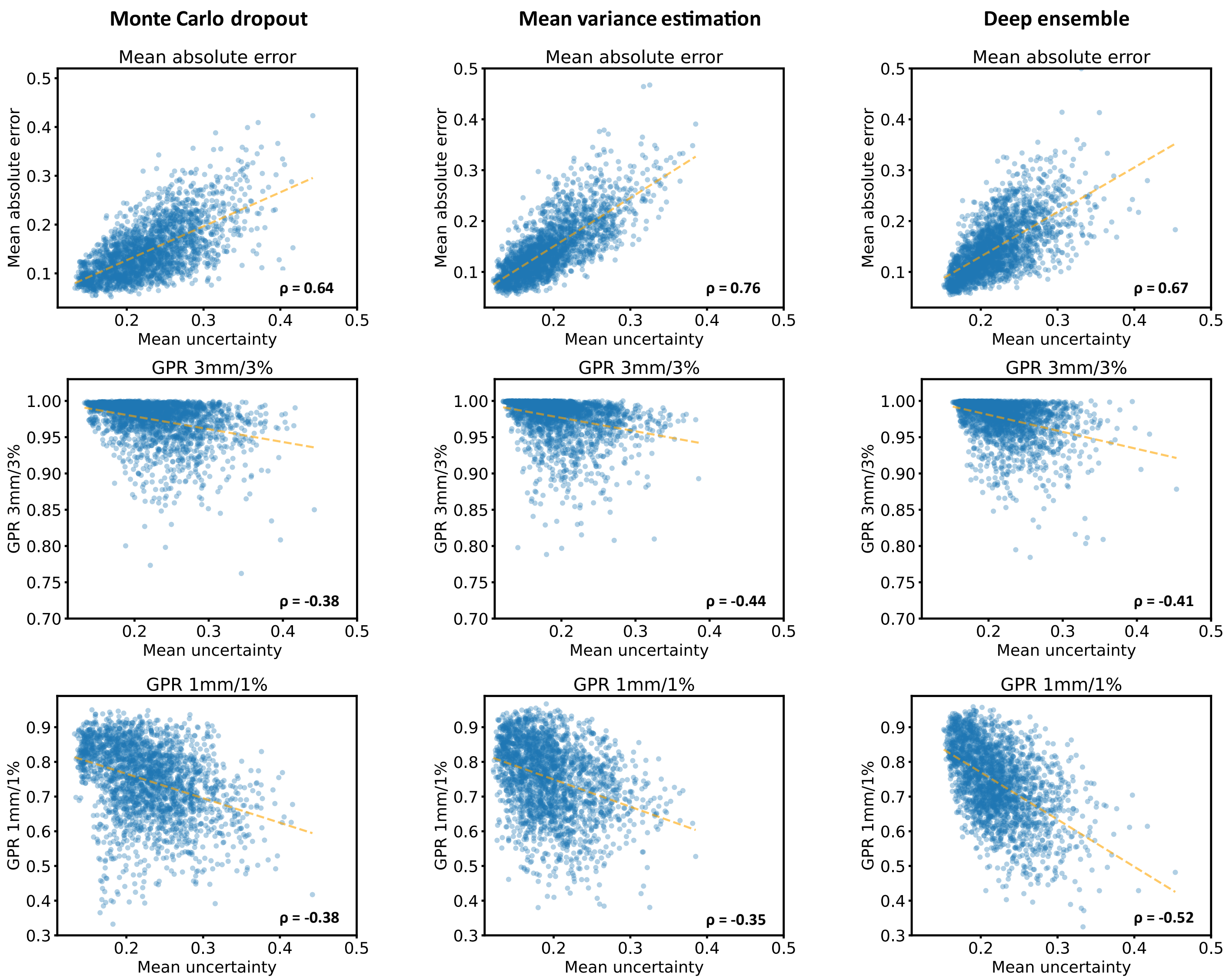

Figure S1: Mean uncertainty per segment against error metrics for the three uncertainty estimation methods, Monte Carlo dropout (left), mean variance Estimation (middle) and deep ensemble (right). GPR 3 mm/3% and GPR 1 mm/1% depict the gamma passing rate per segment with a distance to agreement criterion of 3 mm and 3% dose tolerance and 1 mm and 1%, respectively. The dose cutoff was set to 10% of the maximum dose. A trendline is depicted in orange and Spearman correlation coefficient ρ.
